# The Polarity Protein Scribble Regulates Myelination and Remyelination in the Central Nervous System

**DOI:** 10.1371/journal.pbio.1002107

**Published:** 2015-03-25

**Authors:** Andrew A. Jarjour, Amanda Boyd, Lukas E. Dow, Rebecca K. Holloway, Sandra Goebbels, Patrick O. Humbert, Anna Williams, Charles ffrench-Constant

**Affiliations:** 1 MRC Centre for Regenerative Medicine and MS Society/University of Edinburgh Centre for Translational Research, Scottish Centre for Regenerative Medicine, The University of Edinburgh, Edinburgh, United Kingdom; 2 Cell Cycle and Cancer Genetics Laboratory, Peter MacCallum Cancer Centre, St Andrews Place, East Melbourne, Australia; 3 Max Planck Institute for Experimental Medicine, Göttingen, Germany; 4 Sir Peter MacCallum Department of Oncology, University of Melbourne, Parkville, Victoria, Australia; 5 Department of Biochemistry and Molecular Biology, University of Melbourne, Parkville, Victoria, Australia; 6 Department of Pathology, University of Melbourne, Parkville, Victoria, Australia; Stanford University School of Medicine, UNITED STATES

## Abstract

The development and regeneration of myelin by oligodendrocytes, the myelin-forming cells of the central nervous system (CNS), requires profound changes in cell shape that lead to myelin sheath initiation and formation. Here, we demonstrate a requirement for the basal polarity complex protein Scribble in CNS myelination and remyelination. Scribble is expressed throughout oligodendroglial development and is up-regulated in mature oligodendrocytes where it is localised to both developing and mature CNS myelin sheaths. Knockdown of Scribble expression in cultured oligodendroglia results in disrupted morphology and myelination initiation. When Scribble expression is conditionally eliminated in the myelinating glia of transgenic mice, myelin initiation in CNS is disrupted, both during development and following focal demyelination, and longitudinal extension of the myelin sheath is disrupted. At later stages of myelination, Scribble acts to negatively regulate myelin thickness whilst suppressing the extracellular signal-related kinase (ERK)/mitogen-activated protein kinase (MAP) kinase pathway, and localises to non-compact myelin flanking the node of Ranvier where it is required for paranodal axo-glial adhesion. These findings demonstrate an essential role for the evolutionarily-conserved regulators of intracellular polarity in myelination and remyelination.

## Abstract 2

The polarity protein Scribble regulates the formation and properties of myelin sheaths in the central nervous system during development and after demyelinating injury.

## Author Summary

The formation of myelin, a fatty, multilayered structure that surrounds certain neuronal axons in the nervous system, is essential for the proper communication of electrical signals by neurons, acting both as an insulator and to promote metabolic support to the axon. Loss of myelin can have severe functional consequences and trigger serious diseases, such as multiple sclerosis. Bidirectional communication between the oligodendrocytes, the myelinating cells of the central nervous system, and the axon is essential for the proper formation and function of myelin membranes; however, the signals that control myelination by oligodendrocytes in the central nervous system are poorly understood. In this paper, we use a combination of cell culture and animal studies to demonstrate that the protein Scribble, which is known to be a highly evolutionarily conserved regulator of cell polarity, plays a role in controlling whether oligodendrocytes myelinate axons. We show that Scribble regulates the length and thickness of myelin sheaths formed, as well as the tight adhesion of oligodendroglial membranes to the axonal surface, which is required for the organization of the axon into specialized domains at the nodes of Ranvier (gaps formed between the myelin sheaths generated by different cells). In addition, we show that Scribble plays a key role in the repair of myelin sheaths in a mouse model of demyelinating disease. The discovery of novel regulators of myelination in the central nervous system may allow for the identification of novel therapeutic targets for the promotion of myelin repair in patients suffering from demyelinating diseases.

## Introduction

The myelin sheath, a multilamellar elongation of the plasma membrane formed by oligodendrocytes in the central nervous system (CNS) and Schwann cells in the peripheral nervous system (PNS), allows for the rapid, saltatory conduction of action potentials along axons [1]. In individuals afflicted with demyelinating diseases such as multiple sclerosis (MS), CNS myelin is destroyed, resulting in functional deficits. Endogenous oligodendrocyte precursor cells (OPCs) can migrate into demyelinated lesions, differentiate into oligodendrocytes, and remyelinate damaged regions. However, remyelination eventually fails, resulting in a loss of axonal integrity and irreversible loss of function [2]. Observations from post-mortem analyses of CNS tissue from MS patients suggest that remyelination can fail for several reasons. In some chronic lesions, oligodendrocyte precursor cells do not appear to successfully infiltrate the lesion [3], suggesting a failure of cell migration. In other instances, oligodendroglia populate the lesion but express only early-stage markers, implying a failure of differentiation [4,5]. In other cases, lesions are populated by oligodendrocytes that mature morphologically to varying degrees. This can range from a complete failure of processes to contact axons to the presence of immature oligo-axonal contacts, indicating that these cells have differentiated, but have failed to successfully wrap axons [6].

A critical requirement for myelination is the establishment of intracellular polarity. Following differentiation, oligodendrocytes are first polarized during actin-based nucleation of the nascent process [7]. An actin-based mechanism then results in further extension and branching of oligodendrocyte processes [8]. Oligodendrocyte processes then “sample” their surroundings, forming transient contacts with axons [9]. Some of these contact-forming processes initiate myelin sheath formation by elaborating a sheet of membrane that elongates along and wraps around the axon. During wrapping, the process of compaction—extrusion of the cytoplasm within this membrane sheet—leaves, in the fully-formed sheath, a cytoplasmic channel at the edges of the sheet. Specialised adhesion complexes are formed between the axon and the spiral of uncompacted cytoplasm this channel creates at the end of each sheath—the paranodal loops that flank the small gaps between sheaths called the nodes of Ranvier. The dramatic changes in cell shape required for this essential cell–cell interaction point to a requirement for polarity signalling, and in the PNS, all three highly conserved protein complexes known to establish apico-basal polarity have been implicated in this process. Par-3 interacts with p75 to initiate wrapping by Schwann cells [10], Scribble complex member Discs-large homologue 1 (Dlg1) regulates peripheral myelin initiation and thickness [11,12], while Crumbs complex member Pals1 regulates longitudinal extension of myelinating Schwann cells [13]. However, the signals that control radial and longitudinal extension of myelinating processes in the CNS remain poorly understood. Here we have tested the hypothesis that abnormalities of polarity might cause regeneration failure by examining the expression and function of Scribble in all the different stages of CNS myelination and in remyelination.

## Results

### Scribble Is Expressed in Oligodendroglia and Is Localised to Regions of Axo-glial Contact in CNS Myelin

**Fig 1:**
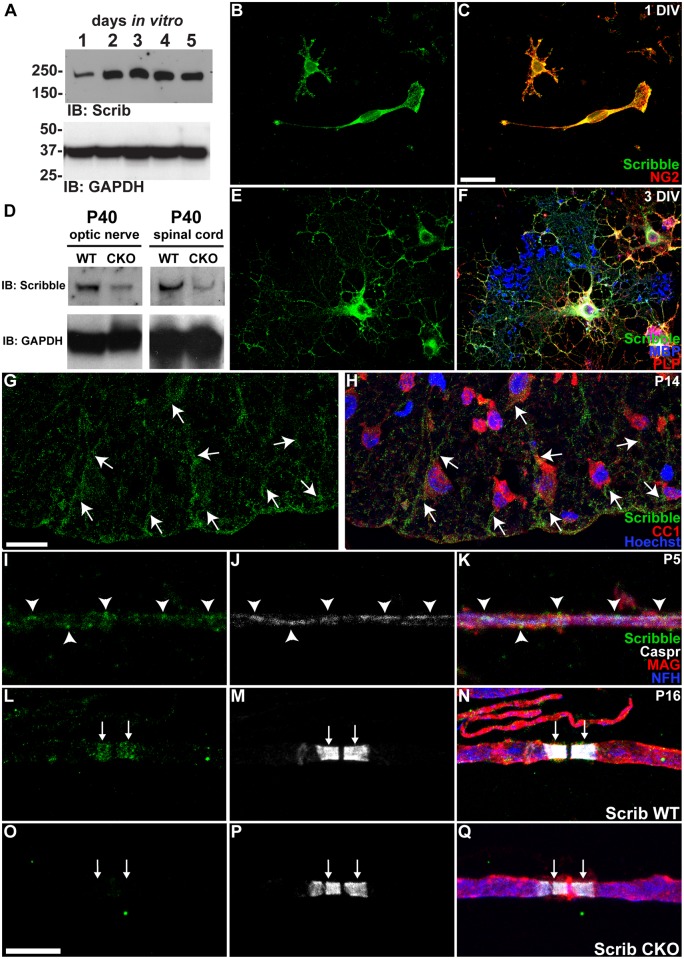
Scribble is expressed by oligodendroglia and is localised to points of axo-glial contact during myelination. A,D; Western blot analysis demonstrated that Scribble protein expression was up-regulated between 1 and 2 DIV, and maintained thereafter. Analysis of CKO tissue (D) confirms the reduction in expression on optic nerve and spinal cord. Glyceraldehyde 3-phosphate dehydrogenase (GAPDH) was used as a loading control. B,C,E,F; Immunocytochemical labelling of cultured oligodendrocyte progenitor cells (B,C) marked by NG2 expression (red), and oligodendrocytes (E,F), marked by PLP and MBP expression (red, blue respectively) express Scribble (green), which is excluded from MBP+ myelin-like sheets. G,H; Immunohistochemical analysis of postnatal day 14 (P14) spinal cord revealed that antibodies against Scribble (green, G,H, arrows) labelled processes of cells recognized by the monoclonal antibody CC1 (red, H), an oligodendrocyte cell body marker. I–Q; Early in myelination, Scribble (green) and Caspr (white) are diffusely localised along the internode (arrowheads) in teased fibre preparations from cervical spinal cords of postnatal day 5 (P5) mice (I–K). Myelin is marked in red by immunolabelling for myelin associated glycoprotein (MAG) and axons in blue by immunolabelling for the 200 kDa neurofilament subunit (NFH). As myelination proceeds, Scribble becomes concentrated at paranodal regions (arrows) in postnatal day 16 (P16) teased fibre preparations (L–N), suggesting that Scribble is concentrated at regions of axo-glial contact during myelination. In mice in which Scribble expression is conditionally eliminated in myelinating glia (Scrib CKO, O–Q), paranodal Scribble immunoreactivity is lost, indicating that Scribble found at CNS paranodes is expressed by oligodendrocytes. Scale bars (B,C,E,F): 25 μm, (G,H): 20 μm (I–Q): 10 μm.

In a previous study aimed at identifying novel targets for remyelinating therapies, gene expression in caudal cerebellar peduncle was characterised following focal demyelination. Among the genes found to be up-regulated at times corresponding to peak oligodendrocyte differentiation and remyelination was the gene coding for the polarity protein Scribble [14]. Although Scribble expression by neurons and astrocytes has previously been reported [15,16], its expression in oligodendroglia has not been characterised. To assess whether Scribble is expressed in oligodendrocytes, we collected lysates from cultured rat oligodendroglia at different stages of differentiation and analysed them by SDS-PAGE and western blotting. Scribble protein expression increased between the first and second day in vitro (DIV) and remained constant thereafter (Fig. 1A), indicating that Scribble expression increases as oligodendrocytes differentiate. Immunocytochemical analyses were used to investigate the localisation of Scribble and revealed that Scribble is localised throughout the cell body and processes of NG2-positive OPCs, and is concentrated at the growth cone-like process tips (Fig. 1B,C). In differentiated oligodendrocytes that express proteolipid protein (PLP) and myelin basic protein (MBP), Scribble is localised in the cell body and processes, but is excluded from MBP-positive myelin membrane sheets (Fig. 1E,F). Scribble is also expressed by CC1-positive oligodendrocytes in vivo, where it is enriched in oligodendrocyte processes (Fig. 1G,H). The localisation of Scribble within myelin was then studied using teased fibre preparations from ventral spinal cord white matter. In preparations from postnatal day 5 (P5) spinal cord, Scribble was diffusely localised throughout the developing myelin sheath (Fig. 1I,K). As myelination progresses, Scribble protein becomes restricted to paranodal regions by P16 (Fig. 1L,N). This pattern of localisation resembles that of the axonal protein Caspr (Fig. 1J,K,M,N), which accumulates at initial points of axo-glial contact before being restricted to paranodes, mirroring the distribution of oligodendroglial neurofascin 155 [17]. To determine whether Scribble is localised to oligodendroglial or axonal membranes, teased fibre preparations were prepared from animals in which Scribble expression had been conditionally eliminated from myelinating glia but not neurons. These mice were obtained by crossing a strain in which loxP sites were inserted into introns 3 and 13 of the *Scribble* gene [18] to a strain in which Cre recombinase is expressed under the control of the 2′3′ cyclic nucleotide 3′ phosphodiesterase [19]. Decreased Scribble protein levels were observed in lysates of optic nerve and spinal cord of Scribble conditional knockout (cKO) animals relative to wild-type (WT) littermates by western blot analysis (Fig. 1D). As expected, this occurred to a greater extent in optic nerve, where oligodendrocytes make up a greater proportion of total cells than they do in spinal cord. In fibres obtained from Scribble cKO mice, Scribble immunoreactivity is absent from paranodes (Fig. 1O-Q), indicating that the protein is oligodendroglial, and not axonal, at paranodes. Taken together, these data suggest that Scribble protein is restricted to non-compact myelin membranes, and its localisation is consistent with a possible role in regulating axo-glial adhesion.

### Scribble Regulates the Morphological Differentiation of Oligodendrocytes

**Fig 2:**
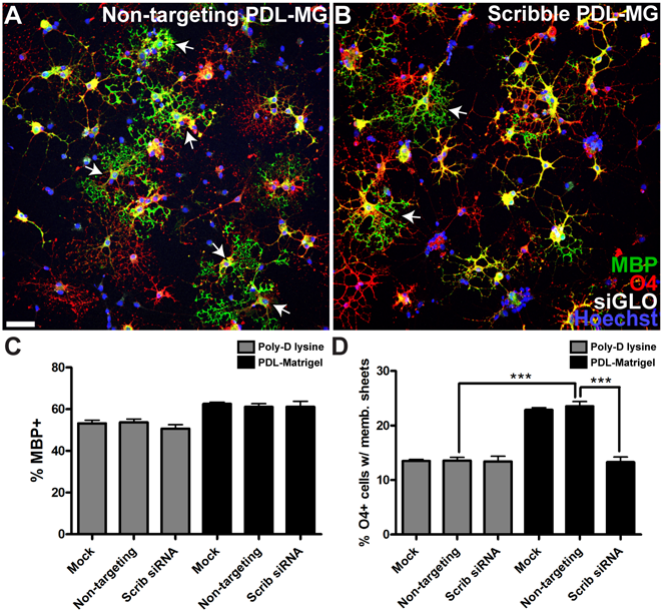
Extracellular matrix-induced myelin sheet formation by oligodendrocytes requires Scribble. A,B; To assess differentiation of OPCs derived from neonatal rat pups into oligodendrocytes, cells were immunolabelled with antibodies against O4 (red) and myelin basic protein (MBP, green). C; Scribble knockdown did not change the proportion of O4-positive cells that express MBP on either poly-D-lysine (PDL, grey bars) or poly-D-lysine-Matrigel (PDL-MG, black bars) substrates. D; Culturing oligodendrocytes on PDL-MG increased the proportion of cells exuding myelin-like membrane sheets (arrows in A). While Scribble knockdown in oligodendrocytes cultured on PDL did not affect the proportion of cells with membrane sheets, the percentage of cells with membrane sheets (arrows in B) was significantly decreased on PDL-MG. Percent of O4-positive oligodendrocytes with myelin membrane sheets: non-targeting PDL = 13.6% ± 0.6%, non-targeting PDL-MG = 23.5% ± 0.8%, Scribble PDL-MG = 13.3% ± 0.9%. Numerical results are presented as mean ± standard error of the mean (SEM). ANOVA with Tukey's multiple comparison test was used, *n* = 6 coverslips per condition, with four fields analysed per coverslip. *** *p* < 0.005. Scale bar = 50 μm.

**Fig 3:**
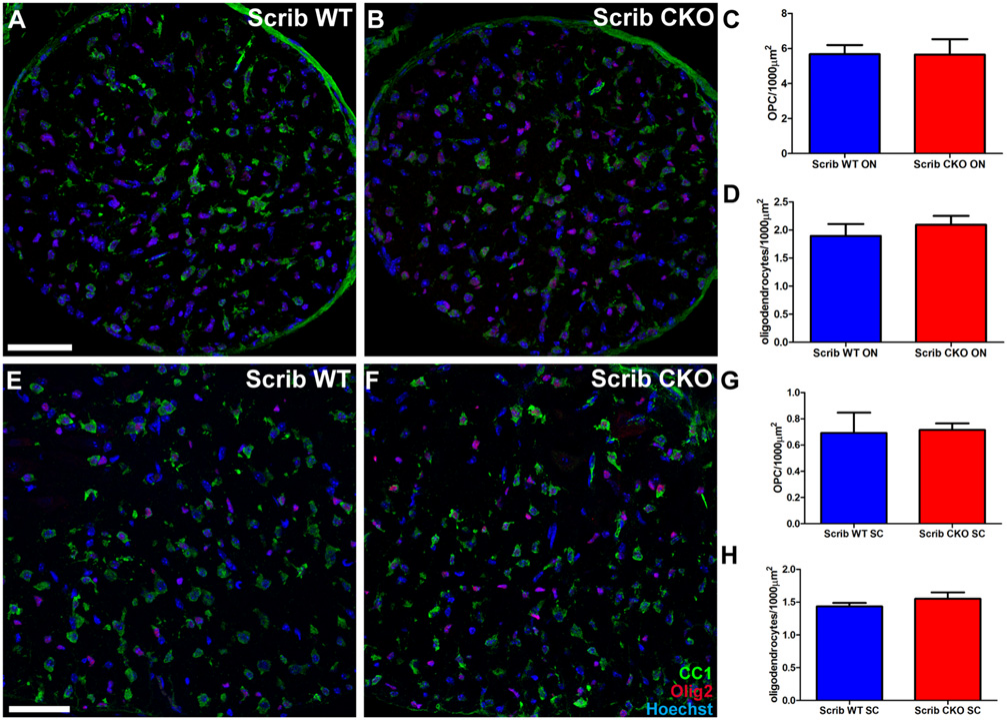
Oligodendroglial differentiation is unaffected in Scribble cKO mice. A–D; The number of OPCs positive for Olig2 (red) and not labelled by antibody CC1 (green), and Olig2 and CC1 double-positive oligodendrocytes present in the optic nerves of wild-type (A) and Scribble CKO (B) did not significantly differ (C,D). E–H; Similarly, the number of OPCs and oligodendrocytes in wild-type (E) and Scribble cKO (F) ventral spinal cord white matter did not differ significantly (G,H). Numerical results are presented as mean ± SEM. Student's *t* test was used, *n* = 4 animals per genotype, at least three fields were analysed per section in at least two non-adjacent sections per animal. Scale bar: 50 μm.

We first investigated potential functional roles for Scribble by studying oligodendrocyte differentiation. This can be split into two distinct processes that enable myelination—expression of myelin proteins and the morphological transformation, manifested by the formation of myelin-like membrane sheets in culture. When oligodendrocytes were cultured on poly-D-lysine and transfected with siRNAs targeting Scribble, we did not observe any change in the proportion of O4-positive cells that express mature oligodendrocyte marker myelin basic protein (MBP, Fig. 2C, grey bars). Similarly, when we examined mice in which Scribble had been conditionally knocked out in oligodendrocytes, the numbers of Olig2+/CC1-oligodendroglial progenitors and CC1+ oligodendrocytes that were present in both Scribble cKO optic nerve (Fig. 3A-D) and ventral spinal cord white matter were unchanged compared to wild-type littermates at P14 (Fig. 3E-H). We conclude that Scribble is not required for the timely expression of myelin proteins. In contrast, however, Scribble does play a role in the second component of differentiation—the morphological transformation that leads to myelin-like membrane sheet formation. This was revealed in experiments asking whether oligodendrocyte differentiation was affected by Scribble knockdown in the presence of extracellular matrix (ECM) cues that promote sheet formation by oligodendrocytes [20]. We used Matrigel as a substrate in these experiments, as it has been demonstrated to localise Scribble protein to basolateral membranes in cultured epithelial cells [21]. As expected, culturing oligodendrocytes on Matrigel increased the proportion of cells with myelin-like membrane sheets (Fig. 2D, black bars). Knockdown of Scribble expression using siRNAs on Matrigel substrates had no effect on the number of MBP-expressing cells (Fig. 2C, black bars) but prevented the increase in cells with myelin-like membrane sheets, showing that Scribble is required for the morphological changes in oligodendrocytes in response to ECM-derived cues. We verified this conclusion using oligodendrocytes derived from Scribble cKO mice and their wild-type littermates. As was observed for rat oligodendrocytes, increased production of myelin-like membrane sheets for cells cultured on Matrigel was observed in wild-type but not Scribble cKO oligodendrocytes (S1 Fig).

### Scribble Is Required for the Initiation of CNS Myelination

**Fig 4:**
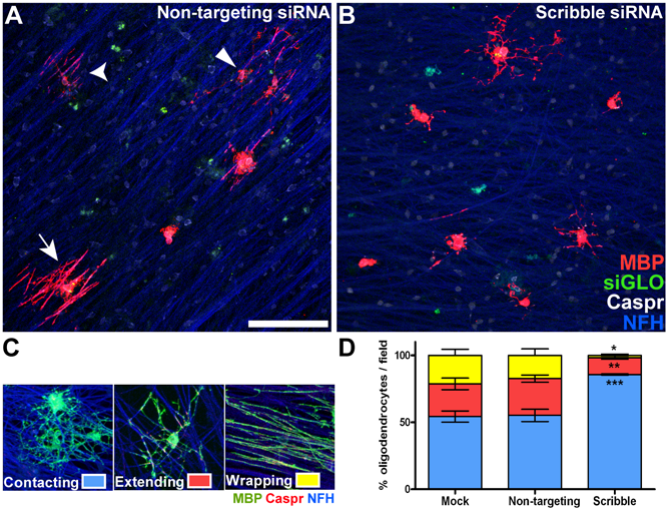
Scribble is required for normal myelin initiation in vitro. A,B; Transfected OPCs were seeded on dorsal root ganglion (DRG) neuron cultures and maintained for 3 d, then immunolabelled with antibodies against MBP (red), Caspr (white), and NFH (blue). MBP-positive oligodendrocytes labelled with siGLO (green) were scored using the categories shown in C as having “contacting” (A, square arrowhead), “extending” (A, curved arrowhead), or “wrapping” morphology (A, arrow). D; Decreased contact and wrapping by MBP-expressing oligodendrocytes was observed in Scribble siRNA-transfected oligodendrocytes (shown in B) relative to those that were mock-transfected or transfected with non-targeting siRNAs (shown in A). Contacting: non-targeting = 55.2% ± 4.6%, Scribble = 85.7% ± 0.4%; extending: non-targeting = 27.4% ± 2.7%, Scribble = 12.6% ± 1.2%; wrapping: non-targeting: 17.4% ± 4.8%, Scribble = 1.7% ± 0.9%. Numerical results are presented as mean ± SEM. ANOVA with Tukey's multiple comparison test was used; *n* = 6 coverslips per condition, five fields were analysed per coverslip. * *p* < 0.05 versus Mock. ** *p* < 0.01 versus Mock, *** *p* < 0.005 versus Mock. Scale bar in A = 100 μm.

Our finding that Scribble is required for ECM-induced morphological differentiation of oligodendrocytes prompted us to investigate the role of Scribble in the different stages of myelination. The first is initiation—the extension along and wrapping around the axon of a myelin sheet formed at the end of oligodendrocyte processes. We first used oligodendrocyte-dorsal root ganglion neuron co-cultures to test whether siRNA knockdown of Scribble influences the ability of oligodendrocytes to initiate extension and wrapping. Three days following addition of oligodendrocytes, just under half the MBP-positive oligodendrocytes were either extending processes along axons or had formed myelin wraps around these axons (Fig. 4A,D; see Fig. 4C for examples of how oligodendrocytes were scored), while in contrast, just under 15% of oligodendrocytes transfected with siRNA targeting Scribble had formed myelin wraps (Fig. 4B,D). Once again, the number of MBP-positive oligodendrocytes did not significantly differ between Scribble knockdown and control conditions, indicating that this was not due to a failure of oligodendrocyte differentiation, but was instead due to the inability of these cells to initiate myelination in the absence of Scribble.

**Fig 5:**
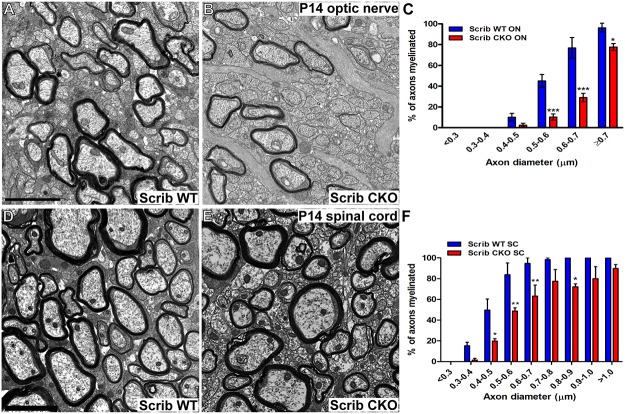
Scribble is required for normal myelin initiation in vivo. A–C; Fewer myelinated axons were observed in P14 optic nerve cross-sections from Scribble conditional knock-out (B) relative to wild-type controls (A). This was observed in axons of all diameters (C; 0.5–0.6 μm: WT = 45.1% ± 6.2%, cKO = 10.3% ± 3.0% myelinated; 0.6–0.7 μm: WT = 76.9% ± 9.9%, cKO = 29.0% ± 4.1% myelinated; ≥0.7 μm: WT = 96.1% ± 3.8%, cKO = 77.7% ± 3.2% myelinated). D–F; Fewer myelinated axons were also observed in P14 spinal cord cross-sections from Scrib cKO (E) relative to wild-type controls (D) for all but the largest-diameter axons (F; 0.4–0.5 μm: WT = 49.7% ± 10.6%, cKO = 19.7% ± 2.3% myelinated; 0.5–0.6 μm: WT = 83.7% ± 11.5%, cKO = 48.7% ± 3.1% myelinated; 0.6–0.7 μm: WT = 94.7% ± 5.3%, cKO = 63.1% ± 10.7% myelinated; 0.8–0.9 μm: WT = 100% ± 0%, cKO = 72.2% ± 2.8% myelinated). Numerical results are presented as mean ± SEM. Two-way ANOVA with Bonferroni's multiple comparisons test was used. *n* = 3 mice per genotype. At least 200 axons analysed per region, per mouse. * *p* < 0.05, ** *p* < 0.01, *** *p* < 0.005 versus Scrib WT. Scale bars in A,E = 2μm.

**Fig 6:**
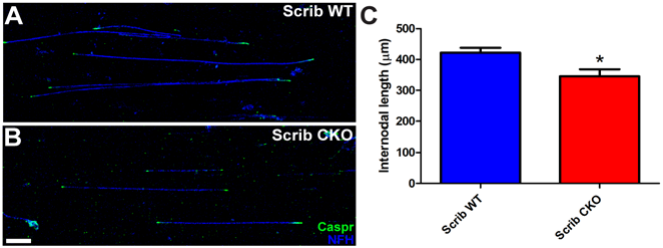
Scribble cKO oligodendrocytes produce shorter myelin internodes. Myelin internodal lengths were determined in teased fibre preparations from P16 Scribble WT (A) and cKO (B) mice by measuring the distance between adjacent Caspr-positive paranodes (green). Internodal length was decreased in Scribble cKO large-diameter spinal cord axons relative to those observed in wild-type littermates (C; Scrib WT: 422.2 ± 16.0 μm, Scrib cKO: 345.8 ± 22.9 μm, *p* = 0.0262). Numerical results are presented as mean ± SEM. Student's *t* test was used; *n* = 3 animals per condition, at least 30 internodes per animal. * *p* < 0.05. Scale bar: 50 μm.

To determine whether Scribble plays a similar role in myelinating oligodendrocytes in vivo, we assessed myelin initiation in the CNS of Scribble cKO mice. As other myelin mutants, such as mice lacking Fyn kinase [22] or laminin α2 [23], have region-specific deficiencies in CNS myelination, we carried out analyses in optic nerve, which mainly consists of smaller-diameter axons, and in the ventral funiculus of the cervical spinal cord, which includes a greater proportion of large-diameter axons. We first performed these analyses at postnatal day 14 (P14), a time of peak myelin formation in both tracts [24]. The percentage of axons lacking any evidence of myelin wrapping in transmission electron micrographs of Scribble cKO optic nerves (Fig. 5B) was greatly increased relative to that seen in wild-type littermates (Fig. 5A) for axons of all diameters (Fig. 5C). Similarly, significantly fewer axons with myelin sheaths were observed in the Scribble cKO spinal cord (Fig. 5E) relative to wild-type (Fig. 5D), though these differences were restricted largely to smaller-diameter axons (<0.7 μm in diameter, Fig. 5F). In addition to revealing fewer axons on which myelin sheaths could be seen, analyses of teased fibre preparations from P16 ventral spinal cord white matter indicated that the nascent sheaths formed by those oligodendrocytes lacking Scribble that had initiated myelination were shorter than those in wild-type oligodendrocytes (Fig. 6), showing that elongation of the myelin sheet along the axon was also impaired.

**Fig 7:**
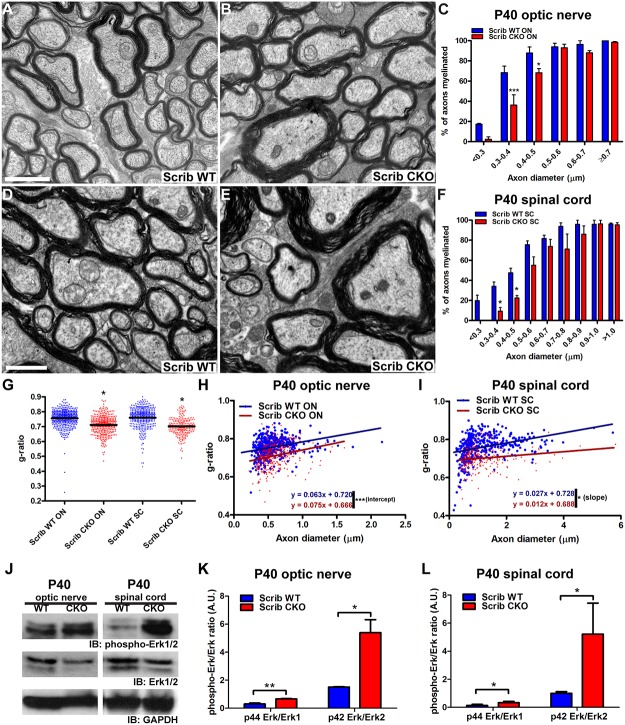
Scribble regulates myelin thickness and ERK activation in oligodendrocytes. A,B,D,E; At P40, decreased myelination of small-diameter axons in both optic nerve (B) and spinal cord (E) of Scribble cKO mice persists relative to wild-type littermates (A,D). This is quantified for optic nerve in C (0.3–0.4μm: WT = 68.4% ± 6.4%, cKO = 36.2% ± 10.1% myelinated, *p* = 0.0002; 0.4–0.5μm: WT = 87.8% ± 6.0%, cKO = 68.2% ± 4.1% myelinated, *p* = 0.0034) and for spinal cord in F (0.3–0.4 μm: WT = 34.0% ± 4.4%, cKO = 9.3% ± 3.7% myelinated, *p* = 0.039; 0.4–0.5μm: WT = 47.5% ± 4.5%, cKO = 22.3% ± 2.5% myelinated, *p* = 0.033) Numerical results here and below are presented as mean ± SEM. Two-way ANOVA with Bonferroni's multiple comparisons test was used. *n* = 3 animals per genotype. At least 200 axons were analysed per animal, per region. G–I; Axons that are myelinated in cKOs have thicker myelin sheaths than do axons of similar diameters in wild-type littermates, as decreased mean g-ratios were observed in both optic nerve and spinal cord (G; optic nerve: WT = 0.755 ± 0.004, cKO = 0.704 ± 0.016, *p* = 0.012; spinal cord: WT = 0.760 ± 0.010, cKO = 0.702 ± 0.008; *p* = 0.018; Student's *t* test was used to compare means from each animal. At least 100 g-ratios were analysed per animal, per region.). The best fit lines obtained by linear regression differed significantly between cKO and wild-type datasets in both optic nerve (H; WT: slope = 0.063 ± 0.012, intercept = 0.720 ± 0.007, cKO: slope = 0.075 ± 0.015, intercept = 0.666 ± 0.010; *p* = 0.548 [slope], *p* < 0.0001 [intercept]) and spinal cord (I; WT: slope = 0.027 ± 0.004, intercept = 0.728 ± 0.006, cKO: slope = 0.012 ± 0.005, intercept = 0.688 ± 0.007; *p* = 0.022 [slope]). J–L; Increased abundance of phosphorylated ERK1/2 relative to total ERK1/2 was observed in western blots of lysates of optic nerve and spinal cord from Scribble conditional mutants (J). The ratio of phospho-ERK/ERK densitometry is provided in arbitrary units for optic nerve (K): p44ERK/ERK1: WT: 0.306 ± 0.064, cKO: 0.666 ± 0.027, *p* = 0.0066; p42ERK/ERK2: WT: 1.511 ± 0.025 cKO: 5.391 ± 0.924, *p* = 0.014; and spinal cord (L): p44ERK/ERK1: WT: 0.127 ± 0.046 cKO: 0.335 ± 0.051, *p* = 0.038; p42ERK/ERK2: WT: 0.997 ± 0.069, cKO: 5.218 ± 1.278, *p* = 0.030, Student's *t* test was used. A,B: Scale bar = 1 um. E,F: Scale bar = 1.5 um. * *p* < 0.05, ** *p* < 0.01, *** *p* < 0.005 versus Scrib WT.

To determine whether this decreased capacity to initiate myelination persisted in older Scribble cKO animals, we repeated these analyses at P40, a time when myelin sheath formation in these regions is complete. By P40, the proportion of mid-sized (0.5–0.7 μm in diameter) and large diameter (≥0.7 μm) axons that are myelinated in Scribble cKO optic nerve (Fig. 7B) and spinal cord (Fig. 7E) does not differ from that observed in wild type (Fig. 7A,D). In both tissues, however, decreased myelination of small-diameter axons (<0.5 μm) is still observed at P40 (Fig. 7C,F), revealing that while myelin initiation catches up in larger diameter axons, the deficits caused by the loss of Scribble are long-lasting in small diameter axons.

### Hypermyelination and Elevated Activation of the ERK MAP Kinase Pathway in Scribble cKO Mice

While analysing the proportion of axons that are myelinated at P40, we observed, paradoxically, that despite the reduced initiation in smaller diameter axons, fully compacted myelin sheaths that were formed in Scribble cKO optic nerve and spinal cord (Fig. 7B,E) appeared thicker than those in wild-type animals (Fig. 7A,D). Quantification of the g-ratio (the ratio of axon circumference to myelin circumference) confirmed this, being decreased in Scribble cKO optic nerve and spinal cord relative to wild-type fibres (Fig. 7G). When this ratio was plotted against axon diameter, lower values were seen for all axon diameters and the equations for the best-fit lines for WT and cKO datasets obtained by linear regression differed significantly in both optic nerve (Fig. 7H) and spinal cord (Fig. 7I). The periodicity of myelin lamellae was unchanged in both optic nerve and spinal cord (S2 Fig), indicating that the decreased g-ratios were due to the presence of more myelin wraps, not increased spacing between them. Importantly, in both P14 and P40 optic nerve and spinal cord, the differences in myelin thickness were not attributable to changes in axon diameter, as their distributions did not differ significantly between wild-type and Scribble cKO animals (S3 Fig).

Previous studies have implicated the ERK/MAP kinase signalling pathway in the regulation of myelin thickness in the CNS, as constitutive activation of this pathway results in aberrantly thick myelin sheaths [25]. Scribble has been reported to negatively regulate the ERK/MAP kinase pathway, as Scribble knockdown results in increased phospho-ERK levels in mammary and skin epithelial cells [26,27]. When lysates from wild-type and Scribble cKO optic nerve and spinal cord were analysed by SDS-PAGE and western blotting, we detected increased levels of phospho-ERK1/2 (Fig. 7J-L), suggesting that the ERK pathway likely plays a role in the hypermyelination that results from loss of Scribble in oligodendrocytes.

### Paranodal Axo-glial Adhesion Is Disrupted in Scribble cKO Mice

**Fig 8:**
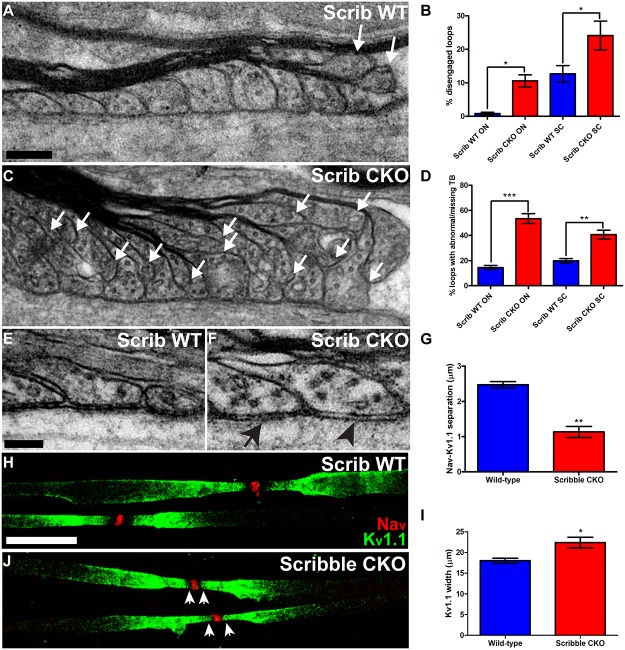
Conditional elimination of Scribble expression in oligodendrocytes disrupts paranodal axo-glial junctions and axonal domain organisation at the node of Ranvier. A–F; In P40 Scribble cKO mice (C), paranodal axo-glial junctions in spinal cord or optic nerve (not shown) displayed an increased proportion of loops that had disengaged from the axonal surface (white arrows) relative to those in the CNS of wild-type littermates (A). This is quantified in B; WT ON: 0.8% ± 0.4%, cKO ON: 10.6% ± 1.8%, WT SC: 12.7% ± 2.4%, cKO SC: 24.1% ± 4.3%. Of the paranodal loops that faced the axolemma, a significantly increased proportion found in Scribble cKO mice were either linked to the axonal membrane by disordered electron density (F, arrow) instead of the ordered transverse bands seen in WT animals (E), or lacked transverse bands entirely (F, arrowhead). This is quantified in D; WT ON: 14.4% ± 1.7%, cKO ON: 53.4% ± 3.9%, WT SC: 19.9% ± 1.6%, cKO SC: 40.7% ± 3.5%. Results were presented as mean ± SEM. Student's *t* test was used. At least 20 nodes of Ranvier were analysed from each of three animals per genotype. G–I; Teased fibre preparations from P40 ventral spinal cord were used to assess myelin domain organisation at nodes of Ranvier in Scribble cKO mice and wild-type littermates. In Scribble cKO nerves, voltage-gated potassium channels normally localised to the juxtaparanode (Kv1.1, green) invade the paranode (J, arrowheads), encroaching on voltage-gated sodium channels at the node of Ranvier (Nav, red), while normal spacing is maintained in wild-type nerves (H), resulting in significantly reduced distances between sodium and potassium channel labelled regions (G; WT: 2.47 ± 0.09 μm, cKO: 1.13 ± 0.15 μm). This is due at least in part to widening of the Kv1.1-immunolabelled domain (I; WT: 18.0 ± 0.6 μm, cKO: 22.4 ± 1.3 μm). Results were presented as mean ± SEM. Student's *t* test was used. At least 30 nodes of Ranvier were analysed from each of four animals per genotype. * *p* < 0.05, ** *p* < 0.01, *** *p* < 0.001. Scale bars: A,C: 200 nm. E,F: 100 nm. H,J: 10 μm.

Next, we examined the final stage of myelin sheath assembly—the formation of axo-glial adhesion complexes between paranodal loops and the axon. Our observation that Scribble localisation becomes restricted to these paranodal loops late in myelination raises the intriguing possibility that this polarity protein has an additional function in regulating paranodal adhesion. In P40 wild-type ventral spinal cord, paranodal loops form orderly contacts with the axon (Fig. 8A). Paranodal junctions in Scribble cKO ventral spinal cord, however, are disorganised with the increased number of paranodal loops (due to the increased number of myelin wraps observed in the CNS) having a decreased association with the axonal surface (Fig. 8B,C). At higher magnification, a ladder-like pattern of electron-dense “transverse bands,” structures that are hypothesised to link the apposed oligodendroglial and axonal membranes together at a fixed distance [28], is visible between the majority of paranodal loops and the axolemma in wild-type CNS (Fig. 8E). These transverse bands were disrupted in paranodal junctions in Scribble cKO CNS (Fig. 8D,F); in some cases, disordered electron density between oligodendroglial and axonal membranes was observed (Fig. 8F, arrow), while in others the bands were completely absent (Fig. 8F, arrowhead). Similar changes to paranode ultrastructure were also observed in P40 optic nerve (not shown, Fig. 8B,D).

As an alternative method of analysing the structural integrity of the paranodal loops, we also examined the gap between the voltage-gated sodium channels responsible for action potential propagation aggregating at the node of Ranvier and the juxtaparanodal *Shaker*-type voltage-gated potassium channels. These two sets of channels are normally separated by the paranodal loops (reviewed by [29–31]). Mislocalisation of potassium channels into the paranodal domain has previously been reported in several mouse mutants where paranodal axo-glial adhesion has been disrupted, including those lacking expression of core junctional components neurofascin 155, caspr, and contactin [32–34]. In teased spinal cord preparations from P40 wild-type mice, a clear gap can be seen between nodal sodium channel (Na_v_) and juxtaparanodal potassium channel subunit (K_v_1.1) immunolabelling (Fig. 8H). At Scribble cKO nodes of Ranvier, this gap is reduced or absent—K_v_1.1 immunolabelling invades the paranodes (Fig. 8J, arrow), widening the K_v_1.1-positive domain (Fig. 8I) and decreasing its separation from nodal Na_v_ immunoreactivity (Fig. 8G). This disorganization does not simply reflect an increased number of paranodal loops, as we found no abnormalities of potassium channel distribution in teased fibre preparations from phosphatase and tensin homologue (PTEN) conditional mutant mice (S4 Fig), which have increased myelin thickness in the CNS [35] and therefore an increase in the number of loops. Taken together, our findings indicate that oligodendroglial expression of Scribble is required for normal paranodal axo-glial adhesion and axolemmal domain organisation in the nodal environs.

### Remyelination Is Impaired in Scribble Conditional Mutants

**Fig 9:**
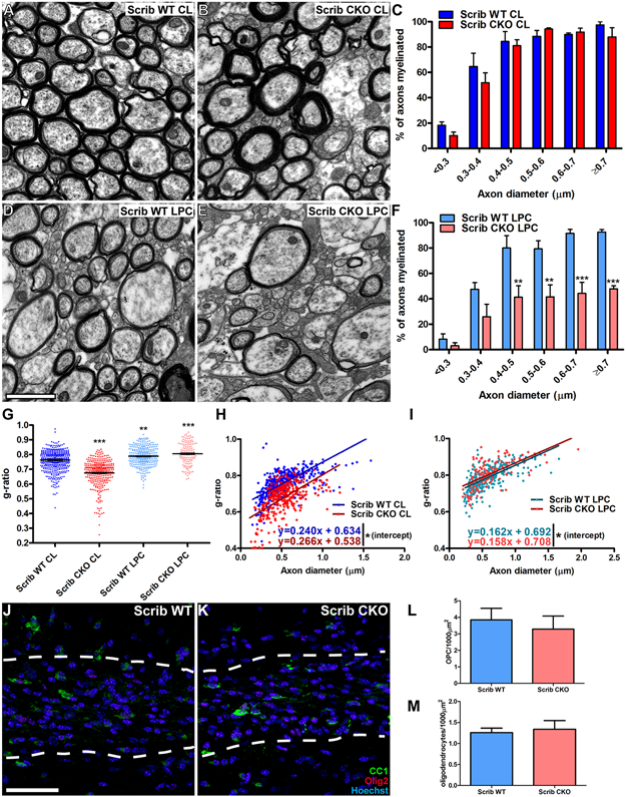
Decreased remyelination initiation is observed in Scribble cKO mice following focal demyelination in corpus callosum. A–F; Focal LPC lesions were induced in corpora callosa of 16-wk-old wild-type and Scribble cKO mice, and myelination was assessed at 21 d following focal demyelination caused by injection with lysophosphatidylcholine (LPC). In the contralateral (unlesioned) hemisphere (CL), no difference was seen in the proportion of axons in the corpus callosum that were myelinated in Scribble cKO compared to wild-type animals (A–C). Fewer remyelinated axons were observed in corpus callosum in the lesion area in Scribble cKO mice (E) relative to controls (D) as quantified in F; 0.4–0.5 μm: WT = 80.0% ± 9.8%, cKO = 41.3% ± 8.9% myelinated; 0.5–0.6 μm: WT = 79.4% ± 6.3%, cKO = 41.5% ± 9.5% myelinated; 0.6–0.7 μm: WT = 91.6% ± 3.0%, cKO = 44.3% ± 8.7% myelinated; >0.7 μm: WT = 92.7% ± 1.9%, cKO = 47.9% ± 2.5% myelinated. Numerical results here and below are presented as mean ± SEM. Two-way ANOVA with Bonferroni's multiple comparisons test was used. *n* = 3 mice per genotype. At least 200 axons were analysed per animal, per region. G–I; Myelin sheaths in the unlesioned hemispheres of Scribble cKO corpora callosa were thicker than those observed in wild-type animals, with both mean g-ratio (G; WT: 0.761 ± 0.010, cKO: 0.675 ± 0.011) and the intercept of the best-fit line (H; WT: slope = 0.240 ± 0.029, intercept = 0.634 ± 0.017, cKO: slope = 0.266 ± 0.022, intercept = 0.538 ± 0.012) being significantly decreased. Following remyelination, mean g-ratios increased significantly as compared to WT contralateral (unlesioned) hemisphere (indicating thinner myelin) and did not differ significantly between wild-type and Scribble cKO remyelinated axons (G), although the Y-intercepts of the best-fit lines obtained by linear regression differed slightly but significantly between cKO and wild-type datasets (I; WT: slope = 0.162 ± 0.011, intercept = 0.692 ± 0.007, cKO: slope = 0.158 ± 0.017, intercept = 0.708 ± 0.012), indicating slightly thinner myelin in Scribble cKO animals. Two-way ANOVA with Bonferroni's multiple comparisons test was used. *n* = 3 mice per genotype. At least 20 g-ratios were analysed per animal, per region. J–M; The density of Olig2+CC1- OPCs (L) and CC1 oligodendrocytes (M) did not differ between lesioned Scribble cKO corpora callosa (K) and those of wild-type animals (J). Student's *t* test. *n* = 3 mice per genotype. * *p* < 0.05, ** *p* < 0.01, *** *p* < 0.001 versus Scrib WT CL. Scale bars: A,B,D,E: 2 μm. J,K: 50 μm.

Finally, we addressed the role of Scribble in remyelination. This was examined by inducing a focal demyelinating lesion in the corpus callosum of young adult (4-month-old) Scribble cKO and wild-type control mice and examining the subsequent remyelination response. Following focal demyelination, fewer remyelinated axons were observed in the corpus callosum of Scribble cKO mice (Fig. 9D, E) at 21 d post-lesion, while in the contralateral (unlesioned) hemisphere no difference in the proportion of myelinated axons was observed (Fig. 9A-C). Interestingly, and in contrast to developmental myelination, the observed decrease in remyelination affected both large- and small-diameter axons (Fig. 9F), indicating that the ability of oligodendrocytes to compensate for the loss of Scribble expression is decreased in the lesion environment. The density of Olig2+CC1- OPCs and CC1+ oligodendrocytes in the lesion areas at 21 d post-lesion is unchanged, indicating that the decreased remyelination in Scribble cKO animals is not due to failed OPC migration or differentiation (Fig. 9J-M).

As in optic nerve and spinal cord, Scribble cKO myelin sheaths in the uninjured (contralateral or CL) corpus callosum were thicker than those in wild-type animals, with the mean g-ratio decreased (Fig. 9G, Scrib cKO CL and Scrib WT CL, respectively) and the linear regression analysis differing significantly (Fig. 9H). G-ratios in the lesioned area following remyelination were increased in both wild-type and Scribble cKO relative to those observed in corpus callosum (Fig. 9G, Scrib WT LPC and Scrib CKO LPC, respectively), consistent with long-established findings in both rodent models and human disease that myelin sheaths formed following remyelination are thinner than those formed during development [36,37]. Interestingly, we saw no evidence for thicker myelin in the remyelinated Scribble cKO corpus callosum when compared to the remyelinated wild-type corpus callosum. The increased myelin thickness seen following development in Scribble cKO oligodendrocytes is therefore not recapitulated following remyelination.

## Discussion

Here, we report four distinct abnormalities following loss of the basal polarity protein Scribble in oligodendrocytes: First, reduced myelin initiation, shown by increased numbers of axons without myelin sheaths. Second, decreased longitudinal myelin extension, indicated by shorter nascent internodes during myelin development. Third, loss of paranodal loop adhesion to the axon, leading to an abnormal distribution of potassium channels normally confined to the juxtaparanode. Finally, increased thickness of myelin in fully developed tracts. These likely reflect differing functions of Scribble in regulating cell signalling, as we shall now discuss.

One striking feature of our findings that may explain how Scribble regulates myelin initiation by oligodendrocytes is that small-diameter axons disproportionately lack myelin sheaths in the Scribble cKO CNS. This resembles our previous finding that in mice expressing a dominant-negative form of β1 integrin, oligodendrocytes require a larger axon diameter in order to initiate myelination normally [38]. Furthermore, our finding here that Scribble is required for the morphological transformation of oligodendrocytes that enables myelination on Matrigel substrates, which contain laminin and other integrin ligands, demonstrates a link between integrin function and Scribble in oligodendrocytes. Polarity proteins regulate the trafficking of proteins to appropriate regions of the cell membrane, with Scribble binding to α5 integrin and diverting it from translocation into lysosomes, thereby increasing its recycling to the cell surface [39]. While α5 integrin is not itself expressed by oligodendrocytes [40], Scribble may stabilize initial adhesions by regulating surface presentation of other integrins or adhesion molecules in response to ECM signals on the axonal surface. Trafficking of other molecules required for myelination may also be directed by basal polarity proteins; for example, in PNS Schwann cells, Dlg1 regulates the localization of exocyst component Sec8, a protein that regulates membrane addition of several myelin proteins in oligodendrocytes [41].

Our finding that lateral elongation of myelinating processes (as measured by the lengths of nascent sheaths) was decreased by Scribble cKO is similar to findings reported for mice in which oligodendrocytes lack the glial isoform of the adhesion molecule neurofascin, NF155 [32]. Both these proteins become restricted to the non-compact regions at the edges of the myelin sheaths that become the paranodal loops, contributing to the axo-glial adhesion complexes of the paranode (as discussed below). This adhesive function in the completed myelin sheath suggests an earlier role in the creation of sufficient traction for the myelinating process of the oligodendrocyte on the axon to elongate, loss or reduction of which would explain the phenotype observed in the Scribble and NF155 cKOs. However, little is known about the signals that control myelin process extension, and a more thorough analysis of the protein complexes at the axo-glial interactions involved is required to test this hypothesis.

The paranodal disruption observed in the Scribble cKOs, with detached loops and spread of potassium channels from the juxtaparanodal region, likely reflects loss of the axo-glial adhesion complexes that anchor the paranodal loops to the axon. Scribble is required for the formation of *Drosophila* septate junctions [42], structures that share ultrastructural and molecular similarity with axo-glial junctions found at paranodes [43], and the loss of transverse bands seen in our electron microscopy analysis shows that these junctions are perturbed in the cKO mice. An alternative explanation, that the paranodal disruption reflects an increased number of loops competing for insufficient space on the axon, is unlikely given the lack of any evidence for paranodal disruption in the PTEN cKO mice whose thicker myelin is, like the Scribble cKO mice, associated with a greater number of loops.

A paradoxical feature of our findings is that loss of Scribble expression in oligodendrocytes reduces myelin initiation and longitudinal extension of the myelin sheath yet increases the final number of wraps, leading to increased myelin thickness. However, several lines of evidence suggest that the number of myelin sheaths formed and myelin sheath thickness are regulated independently in the CNS. Mice lacking ERK1/2 or fibroblast growth factor (FGF) receptors show defects in myelin thickness but not initiation [44,45], while the thickness of myelin sheaths formed by oligodendrocytes lacking β1 integrin function is unchanged [38]. A similar role in the positive regulation of initiation and negative regulation of myelin thickness has been reported for Dlg1 in Schwann cells [11,12]. However, unlike Scribble in the CNS, Dlg1 appears to reduce myelin formation by Schwann cells by inhibiting Akt activation through an interaction with PTEN. Scribble, by contrast, appears to act by inhibiting the ERK pathway in oligodendrocytes (Fig. 6). These differences may be attributable to intrinsic differences between signalling in myelinating oligodendrocytes and Schwann cells as, for instance, neuregulin 1 is required for myelination in PNS [46], whilst its role in CNS is one of modulation [47]. One possible mechanism by which Scribble negatively regulates ERK activation in oligodendrocytes is through the recruitment of protein phosphatase 1 (PP1). Scribble associates directly with the PP1 catalytic subunit, an interaction that may underlie the role of Scribble as a tumour suppressor [48,49]. Whilst little is known about the role of PP1 in regulating CNS myelination, analysis of the transcriptome during oligodendrocyte differentiation indicates that the inhibitory PP1 regulatory subunit PPP1r14a is highly up-regulated during differentiation [50], suggesting that PP1 inhibition may be important for CNS myelination. Additionally, PPP1r14a was found to be highly up-regulated during remyelination in our earlier microarray analysis [14], suggesting that regulation of PP1 activity may also be important for myelin repair.

Less well understood at present is the relationship between myelin thickness and internodal length in the CNS. Whilst both correlate positively with increasing axon diameter [51], our finding that Scribble simultaneously promotes longitudinal extension of the myelin membrane along the axon while inhibiting radial extension shows clearly that the molecular machinery responsible for each is distinct. Interestingly, this observation is consistent with a recent report suggesting that the extension of myelin membrane in the CNS is the result of two distinct motions: lateral extension of the outermost cytoplasm-filled edges of each myelin layer, which directly contact the axon and later align to form the paranodal loops, and a “growth zone” at the inner tongue where new myelin membrane is formed [52]. One possibility is that Scribble function is “compartmentalised” through interactions with different protein complexes at sites of radial or longitudinal growth.

Given the roles that we have demonstrated for Scribble in morphological differentiation of oligodendrocytes, myelin initiation, and myelin thickness regulation, the finding that it also plays a critical role in myelin regeneration is not surprising. However, our finding that the initiation of myelination is more severely disrupted during remyelination than during development in Scribble cKO mice indicates that loss of polarity signalling may have more significant consequences for myelinating oligodendrocytes in an injured environment. Several studies have demonstrated up-regulation of ECM proteins in demyelinated lesions, both in rodent models [53] and in multiple sclerosis patient tissue [54]. Further study of the interactions between ECM signalling and the function of polarity proteins following demyelination could therefore allow for the further identification of signalling pathways that are of particular importance for myelin repair.

While our study represents the first evidence for a role of basal polarity proteins in CNS myelination by oligodendrocytes, previous work has identified key roles in PNS myelination by Schwann cells. However, there may be important differences in the patterns of polarity between the two cell types. Previous studies have shown that when proteins destined for compact myelin in oligodendrocytes, such as PLP, are expressed in polarised epithelial cells, they are sorted to apical membranes [55]. In contrast, proteins normally targeted to non-compact myelin regions, such as the paranodal protein NF155, become localised to basal or lateral membranes [56]. This has led to the view that the myelin sheath in the CNS is a polarized structure, with compact myelin being an apical membrane and non-compact membranes such as paranodal loops (in which Scribble is localized) having the characteristics of basolateral membranes (reviewed by [57]). In the PNS, however, it is the apical Crumbs complex protein Pals1 that is enriched in paranodes (where it positively regulates myelin thickness and internodal length), while Scribble is localised primarily on the outer part of the sheath and does not appear to be enriched at paranodes [13]. While the localisation and function of Pals1 and other Crumbs complex proteins has not yet been established in oligodendrocytes, the difference in Scribble localisation suggests that important differences exist between the roles played by polarity proteins in CNS and PNS myelin.

## Materials and Methods

### Animals

All animal work conformed to United Kingdom legislation Animals (Scientific Procedures) Act 1986 and to the University of Edinburgh regulations. For terminal procedures, animals were killed with an overdose of Euthatal. For focal demyelinating lesion experiments, animals were anesthetised with isofluorane. Vetergesic and Rimadyl were used to relieve pain following surgery. Mice in which the *Scribble* gene was targeted for conditional elimination were generated by insertion of a 5′LoxP site into intron 3 and a 3′LoxP site into intron 13, as described previously [18]. Mice in which the PTEN gene was similarly targeted were generated by inserting LoxP sites into exon 5 [58]. To conditionally eliminate *Scribble* expression in myelinating glia, Scribble^fl/fl^ animals were crossed with animals expressing Cre recombinase under the control of the 2′–3′ cyclic nucleotide phosphodiesterase (CNP) gene [19].

### Antibodies and Protein Conjugates

Primary antibodies used for rodent experiments were rabbit anti-Caspr (Abcam, UK), mouse CC1 monoclonal antibody (Abcam, UK), rabbit anti-GAPDH (Sigma, UK), mouse anti-myelin-associated glycoprotein (MAG, Millipore, UK), rabbit anti-Kv1.1 (Abcam, UK), rat anti-myelin basic protein (MBP, Serotec, UK), chicken anti-neurofilament heavy chain (NFH, Encor Biotechnologies, Florida, United States), rabbit anti-Olig2 (Sigma, UK), mouse anti-O4 (Immunosolv, UK), mouse anti-NG2 (Millipore, UK), mouse anti-proteolipid protein (PLP, Millipore, UK), goat anti-Scribble (C20, Santa Cruz Biotechnology, US), mouse anti-pan sodium channel (Sigma, UK). Secondary antibodies used were Alexa 488-conjugated goat anti-rabbit IgG, Alexa 488-conjugated donkey anti-goat IgG, Alexa 568-conjugated donkey anti-rat IgG, Alexa 568-conjugated donkey anti-mouse IgG, and Alexa 568-conjugated goat anti-rabbit IgG (all from Invitrogen, UK), DyLight 405-conjugated donkey anti-rat IgG, and DyLight 405-conjugated donkey anti-chicken IgG (Stratech, UK).

### Rat Oligodendroglial Cultures

OPC cultures were prepared by shaking-off from mixed glial cultures prepared from neonatal Sprague Dawley rat cortices as described previously [59]. OPCs were seeded on PDL-coated 18 mm glass coverslips and maintained in SATO medium with 0.5% FCS at 37°C in 7.5% CO_2_. For analyses of protein expression, oligodendroglia cultured for 1–5 d in vitro were lysed for 10 min on ice with TEN buffer with 1% Triton-X-100 and 1X protease and phosphatase inhibitor cocktails (Calbiochem) and then subjected to SDS-PAGE and western blotting. For immunocytochemical analyses, cultures were fixed with 4% paraformaldehyde and washed twice in PBS and blocked with blocking solution (4% heat-inactivated donkey serum, 2% BSA, and 0.1% Triton X-100 in PBS). Cultures were incubated overnight in primary antibodies diluted in blocking solution. Following repeated washes with PBS, cultures were incubated with secondary antibodies, washed with PBS, and then mounted. Confocal z-stacks were acquired using a Leica SPE inverted confocal microscope (63x objective, zoom 1), and images were processed using Image Pro Plus (Media Cybernetics, UK) and Photoshop (Adobe, US) software. OPC surface area and diameter were measured using Image Pro Plus. Oligodendrocyte differentiation was analysed by scoring cells as having either “simple” (short, non-interdigitating processes), “complex” (longer, interdigitating processes, but not membrane sheets), or “membrane” morphology (processes containing MBP-positive membrane sheets). Five 20x fields from each of six coverslips were analysed per condition. At least two independent experiments were performed for all analyses.

### Mouse Oligodendroglial Cultures

Cortices from individual P9 mice were processed to single-cell suspensions using the gentleMACs dissociator (Miltenyi Biotec), according to the manufacturer's instructions. Mouse OPC cultures were prepared from cell suspensions by immunopanning with anti–platelet-derived growth factor (PDGF)alpha receptor antibody as described previously [60]. OPCs from each animal were maintained separately under proliferating conditions in T-75 flasks for 7–9 d, de-adhered from flasks using TrypLE (Life Technologies), and seeded on 13 mm glass coverslips. Following differentiation into oligodendrocytes for 2 d, cultures were fixed and analysed immunocytochemically. Immunocytochemical analyses, imaging, and quantification were performed as described above for rat oligodendrocytes.

### Immunohistochemical Analysis

Mice were anaesthetised and perfused with 4% paraformaldehyde in PBS. Brains, spinal cords and/or optic nerves were postfixed with 4% paraformaldehyde overnight, equilibrated in 15% and then 30% sucrose solutions in PBS, and then embedded in Optimal Cutting Temperature compound (VWR, UK). Cryosections of 10 μm were cut and mounted onto Superfrost Plus slides (Thermo Fisher, UK). Tissue sections were immunolabelled as described above for cultures. Confocal z-stacks were acquired using a Leica SP8 inverted confocal microscope (40x objective, zoom 1), and images processed using Image Pro Plus (Media Cybernetics, UK) and Photoshop (Adobe, US) software. For analyses of oligodendroglial cells in CNS white matter, Olig2+CC1- OPCs and CC1+ oligodendrocytes were counted and these values divided by the area of the region in which the counts were performed to obtain a density value expressed as cells per 1,000 μm^2^. For optic nerves, cells in the entire nerve section were counted, while in spinal cord sections only cells present in white matter areas were included. At least two non-adjacent sections from each of four animals were analysed per genotype.

### Teased Spinal Cord Fibres

Postnatal day 5 or 16 mice were anaesthetised and perfused with 4% paraformaldehyde in PBS. Spinal cords were then postfixed with 4% paraformaldehyde for 30 min. Fibres from the ventral funiculus of cervical spinal cord were peeled off and cut into approximately 3 mm by 1 mm strips. Strips were placed onto SuperFrost Plus slides (Fisher, UK) and gently teased apart using acupuncture needles (Journal of Chinese Medicine, UK). Immunohistochemical labelling was carried out as described above for cultures. For analyses of axonal domains at paranodes, the shortest distance along the axon between the edge of the Na_v_-positive domain and Kv1.1-positive domain was measured. When these domains contacted each other or overlapped, the distance was measured as zero. For measurements of the Kv1.1-positive domain, the distance measured was the maximal distance along the axon where continuous immunoreactivity could be observed.

### siRNA Transfections

For oligodendrocyte-DRG co-culture experiments, oligodendrocytes were transfected twice on consecutive days prior to shake-off from mixed glial cultures. Prior to transfection, medium was removed and replaced with DMEM with 10% foetal bovine serum (FBS) without antibiotics, and cells transfected with 20 μM siRNA (or mock transfected) and 20 μM siGLO transfection indicator using 1% Lipofectamine RNAiMAX transfection reagent (Invitrogen) in OPTI-MEM. Following the second transfection and prior to shake-off, medium was replaced with DMEM with 10% FBS and pen-strep (Invitrogen). For studies of oligodendrocyte morphology, transfections were carried out following shake-off. OPCs were maintained in SATO medium with pen-strep, 0.5% FBS and 10 nM PDGF and FGF overnight. Medium was removed and replaced with SATO with 0.5% FBS, and cells transfected with 20 μM siRNA (or mock transfected) using 1% Lipofectamine RNAiMAX transfection reagent (Invitrogen) in OPTI-MEM. siRNA sequences used were obtained from Dharmacon/Thermo Scientific (Scribble: L-080977, Non-targeting: D-001810, siGLO: D-001630). Medium was replaced with SATO with 0.5% FBS and pen-strep after 6 h. Cells were maintained in culture for 48–72 h, and then fixed or lysed as described above. In all experiments, knockdown of protein expression was verified by SDS-PAGE and western blotting.

### Myelinating Rat OPC-DRG Neuron Co-cultures

Rat OPC-DRG co-cultures were prepared as described previously [14]. Cultures were fixed and processed for immunocytochemistry as described above for oligodendrocyte cultures. Oligodendrocytes were scored for their morphology as “contacting” (processes touching but not aligning with axons), “extending” (processes aligned with, but not surrounding axons), or “wrapping” (MBP and Caspr-positive internodes clearly visible). Six 20x fields from each of four coverslips were analysed per condition.

### Focal Demyelination of Mouse Corpora Callosa

Focal demyelination experiments were performed using 16-wk-old wild-type and Scribble cKO mice, as described previously [61].

### Transmission Electron Microscopy

Samples were fixed in 4% paraformaldehyde/2.5% glutaraldehyde in 0.1 M phosphate buffer, pH 7.3, for 2 h; postfixed in 1% glutaraldehyde in 0.1 M phosphate buffer for 14 d; washed in PBS; and postfixed in 1% Osmium Tetroxide in 0.1 M phosphate buffer for 60 min. The samples were then dehydrated in increasing concentrations of ethanol and embedded in Araldite resin. Sections, 1 μm thick, were cut on a Reichert OMU4 ultramicrotome (Leica Microsystems), stained with Toluidine Blue, and viewed in a light microscope to select suitable areas for investigation. Ultrathin sections of 60 nm were cut from selected areas, stained in Uranyl Acetate and Lead Citrate, and then viewed in a Phillips CM120 Transmission electron microscope (FEI). Images were taken on a Gatan Orius CCD camera (Gatan). G-ratio was analysed in coronal sections of spinal cord and optic nerve, and was determined by dividing axon diameter by the total diameter of the axon and overlying myelin sheath. Axon diameter was calculated from measured axon perimeter based on an assumption of circularity. For measurements taken from optic nerve, the diameter of the myelinated axon was determined similarly. For measurements of g-ratio in spinal cord, where myelin sheaths often appeared uncompacted, myelin sheath thickness was measured directly at the point at which the myelin sheath was most compact, and myelinated axon diameter was determined by adding this value to the calculated axon diameter. Analyses of paranodal structure were performed using sagittal sections of optic nerve and ventral spinal cord white matter. All measurements were performed using Image Pro Plus software (Media Cybernetics). For analyses of the proportion of axons myelinated and g-ratio, at least 200 axons per animal were analysed, while paranodal ultrastructure was analysed in at least 20 nodes of Ranvier per animal. All analyses were carried out in three animals per genotype.

## Supporting Information

S1 Data Excel spreadsheet containing, in separate sheets, the underlying numerical data for figure panels 2C, 2D, 3C, 3D, 3G, 3H, 4D, 5C, 5F, 6C, 7C, 7F, 7G, 7H, 7I, 7K, 7L, 8B, 8D, 8G, 8I, 9C, 9F, 9G, 9H, 9I, 9L, 9M, S1C, S1D, S2C, S3A, S3B, S3C, S3D, and S4C. (XLSX)

S1 Fig Conditional elimination of Scribble in mouse oligodendrocytes reproduces the effect of siRNA elimination of Scribble expression in rat oligodendrocytes. A,B; OPC cultures from Scribble cKO mice and their wild-type littermates were seeded on poly-D-lysine (PDL) or, as shown, poly-D-lysine-Matrigel (PDL-MG) coated coverslips and differentiated for two days. To assess differentiation, cells were immunolabelled with antibodies against O4 (red) and myelin basic protein (MBP, green). C; Conditional elimination of Scribble did not change the proportion of O4-positive cells that express MBP on either PDL (grey bars) or PDL-MG (black bars) substrates. D; Culturing oligodendrocytes on PDL-MG increased the proportion of cells exuding myelin-like membrane sheets. While elimination of Scribble expression in oligodendrocytes cultured on PDL did not affect the proportion of cells with membrane sheets, the percentage of cells with membrane sheets was significantly decreased on PDL-MG compared to what was observed for wild-type oligodendrocytes on PDL-MG. Percent of O4-positive oligodendrocytes with myelin membrane sheets: Scrib WT PDL = 41.1% ± 1.8%, Scrib WT PDL-MG = 51.2% ± 1.5%, Scribble cKO PDL-MG = 37.1% ± 1.6%. Numerical results are presented as mean ± SEM. ANOVA with Tukey's multiple comparison test was used. *n* = 4 animals were used per condition, five fields from each of three coverslips per animal were analysed. ** *p* < 0.01, *** *p* < 0.001. Scale bar = 50 μm.
(TIF)

S2 Fig Compact myelin periodicity is unchanged in Scribble cKO mice. The mean distance between major dense lines (the periodicity) in P40 optic nerve (not shown) and ventral spinal cord compact myelin (A,B) was unchanged in Scribble cKO mice compared to wild-type littermates (C). The effect of lost Scribble expression in oligodendroglia on g-ratio was, therefore, not due to changes of spacing between compact myelin lamellae. At least 15 axons per tissue from each of three animals per genotype were analysed. Numerical results are presented as mean ± SEM. Student's *t* test was used. Images were obtained at 37000x. Scale bar = 20 nm.
(TIF)

S3 Fig Knockdown of Scribble in oligodendroglia does not influence axon diameter in the central nervous system. No change in the Scribble cKO mice is seen in the proportion of axons with diameters of less than 0.4 μm, between 0.4 and 0.6 μm, or greater than 0.6 μm in optic nerve (A,C) or axons with diameters of less than 0.5 μm, between 0.5 and 0.8 μm, or greater than 0.8 μm in ventral spinal cord (B,D). The distribution of axon diameters observed was unchanged at both P14 (A, B) and P40 (C,D). Numerical results are presented as mean ± SEM.
(TIF)

S4 Fig Axonal domain organisation at the node of Ranvier is unaffected in PTEN cKO mice. In teased ventral spinal cord preparations from both P40 PTEN cKO mice (B) and wild-type littermates (A), voltage-gated potassium channels were normally localised to the juxtaparanode (Kv1.1, green), with no change in the separation by paranodal junctions from voltage gated sodium channels at the node of Ranvier (Nav, red), indicating the paranodal barrier separating them remains intact (C). Numerical results are presented as mean ± SEM. At least 20 paranodes were analysed in five mice per genotype. Student's *t* test was used. Scale bar = 20 μm.
(TIF)

## Abbreviations

APC: Adenomatous polyposis coli
cKO: conditional knockout
CNS: central nervous system
DIV: day in vitro
Dlg1: Discs-large homologue 1
DRG: dorsal root ganglion
ECM: extracellular matrix
ERK: Extracellular signal-related kinase
FGF: fibroblast growth factor
GAPDH: Glyceraldehyde 3-phosphate dehydrogenase
MAG: myelin associated glycoprotein
MAP kinase: mitogen-activated protein kinase
MBP: myelin basic protein
MS: multiple sclerosis
NFH: neurofilament subunit
OPC: oligodendrocyte progenitor cell
P5: postnatal day 5
PDL: poly-D-lysine
PDGF: platelet-derived growth factor
PLP: proteolipid protein
PNS: peripheral nervous system
PP1: protein phosphatase 1
PTEN: phosphatase and tensin homologue
WT: wild-type
