# Supplementary figures and images for: The Polarity Protein Scribble Regulates Myelination and Remyelination in the Central Nervous System
Source: PLoS Biol. 2015 Mar 25;13(3):e1002107. doi: 10.1371/journal.pbio.1002107 (PMC4373955; doi:10.1371/journal.pbio.1002107)

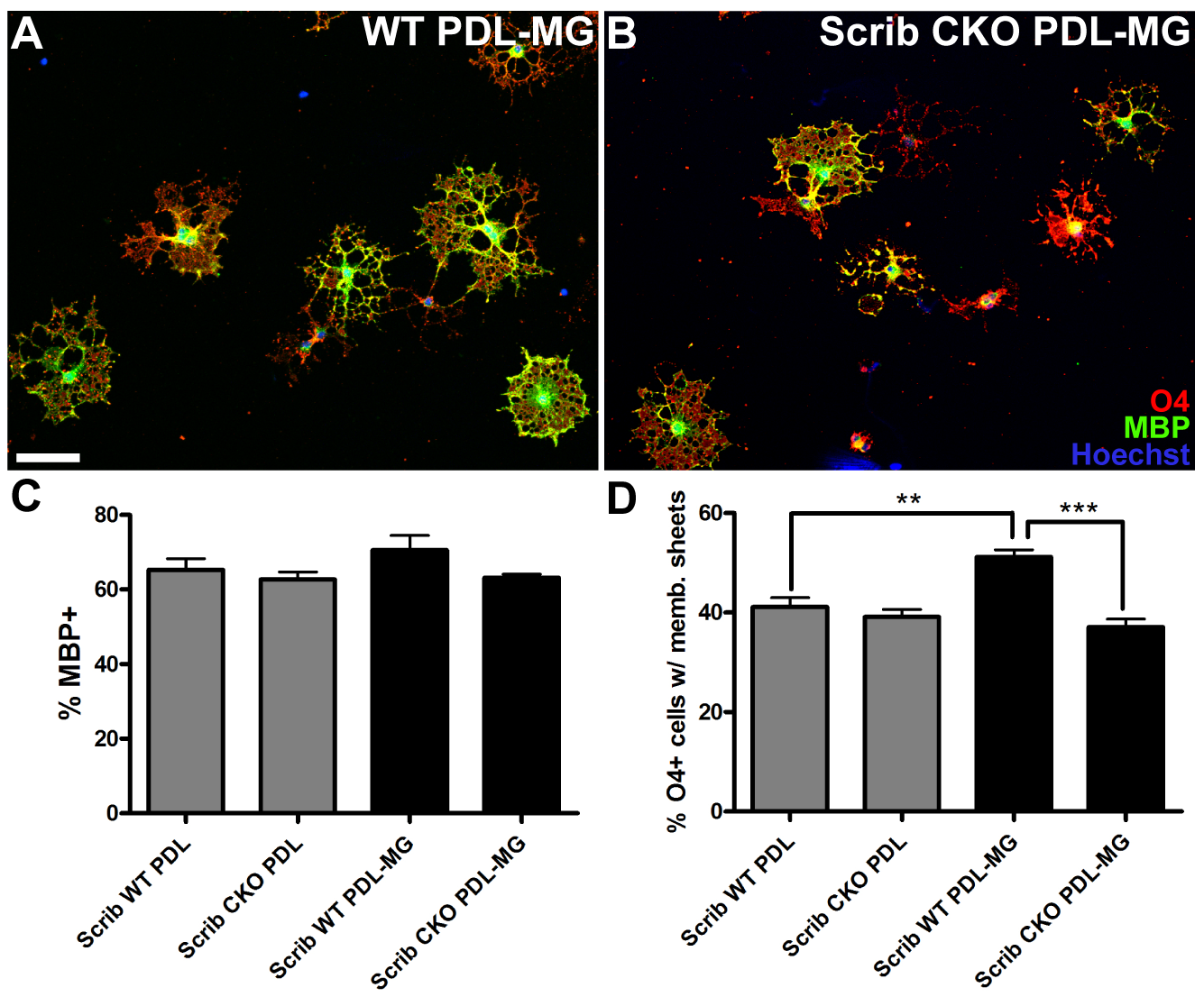

Supplement: S1 Fig — A,B; OPC cultures from Scribble cKO mice and their wild-type littermates were seeded on poly-D-lysine (PDL) or, as shown, poly-D-lysine-Matrigel (PDL-MG) coated coverslips and differentiated for two days. To assess differentiation, cells were immunolabelled with antibodies against O4 (red) and myelin basic protein (MBP, green). C; Conditional elimination of Scribble did not change the proportion of O4-positive cells that express MBP on either PDL (grey bars) or PDL-MG (black bars) substrates. D; Culturing oligodendrocytes on PDL-MG increased the proportion of cells exuding myelin-like membrane sheets. While elimination of Scribble expression in oligodendrocytes cultured on PDL did not affect the proportion of cells with membrane sheets, the percentage of cells with membrane sheets was significantly decreased on PDL-MG compared to what was observed for wild-type oligodendrocytes on PDL-MG. Percent of O4-positive oligodendrocytes with myelin membrane sheets: Scrib WT PDL = 41.1% ± 1.8%, Scrib WT PDL-MG = 51.2% ± 1.5%, Scribble cKO PDL-MG = 37.1% ± 1.6%. Numerical results are presented as mean ± SEM. ANOVA with Tukey's multiple comparison test was used. n = 4 animals were used per condition, five fields from each of three coverslips per animal were analysed. ** p < 0.01, *** p < 0.001. Scale bar = 50 μm. (TIF) [file pbio.1002107.s002.tif]

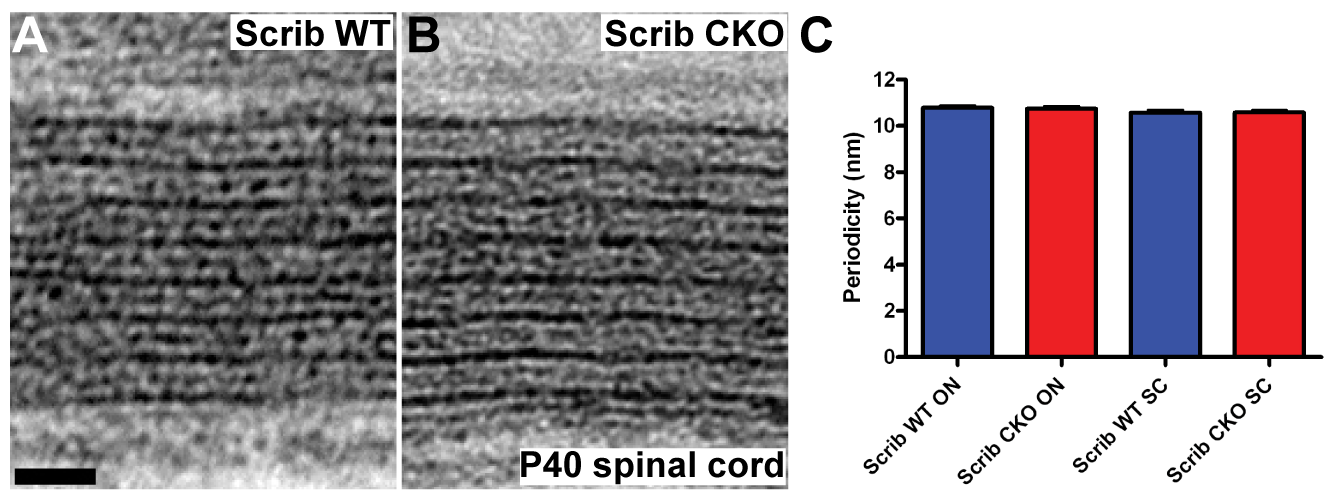

Supplement: S2 Fig — The mean distance between major dense lines (the periodicity) in P40 optic nerve (not shown) and ventral spinal cord compact myelin (A,B) was unchanged in Scribble cKO mice compared to wild-type littermates (C). The effect of lost Scribble expression in oligodendroglia on g-ratio was, therefore, not due to changes of spacing between compact myelin lamellae. At least 15 axons per tissue from each of three animals per genotype were analysed. Numerical results are presented as mean ± SEM. Student's t test was used. Images were obtained at 37000x. Scale bar = 20 nm. (TIF) [file pbio.1002107.s003.tif]

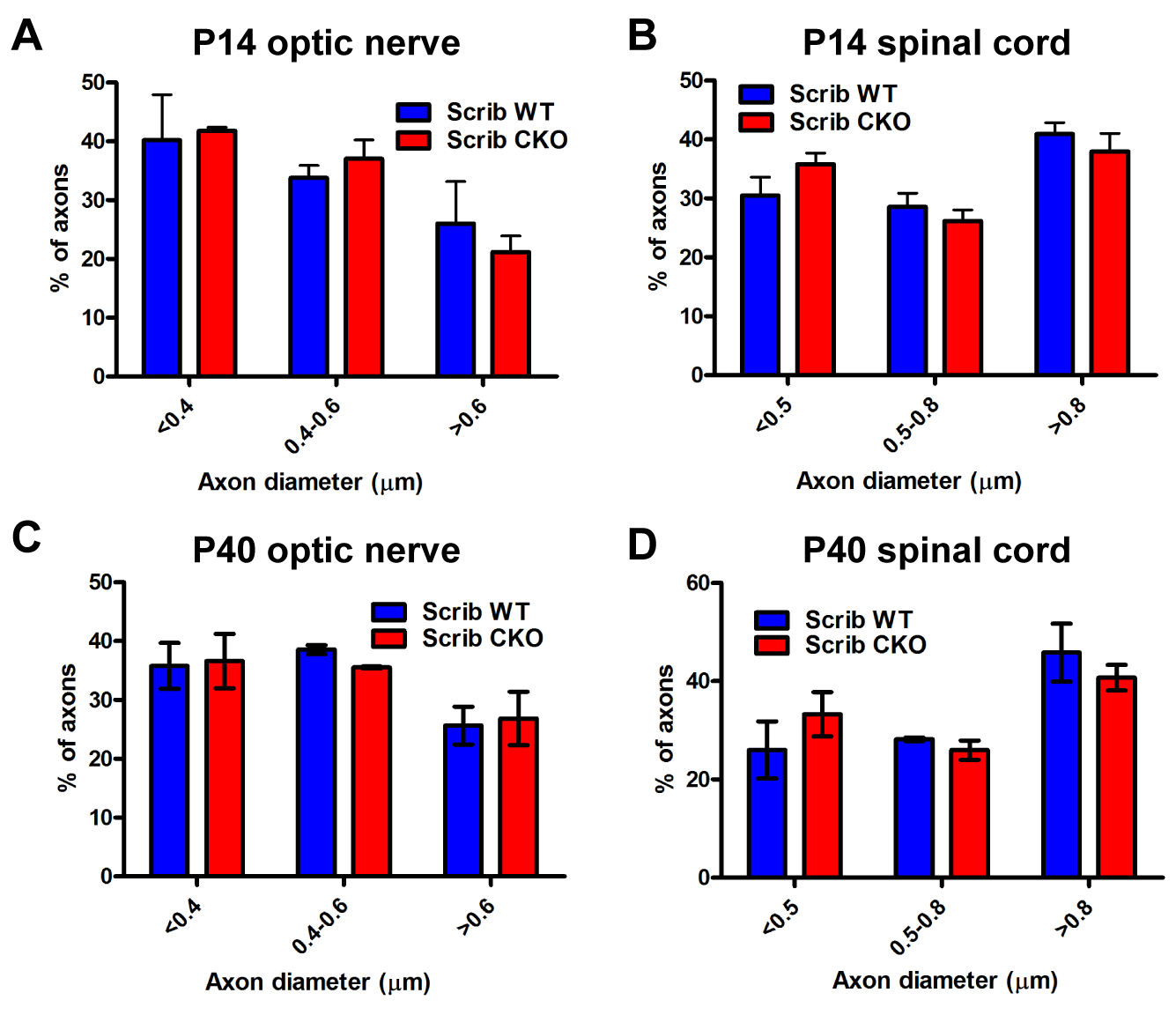

Supplement: S3 Fig — No change in the Scribble cKO mice is seen in the proportion of axons with diameters of less than 0.4 μm, between 0.4 and 0.6 μm, or greater than 0.6 μm in optic nerve (A,C) or axons with diameters of less than 0.5 μm, between 0.5 and 0.8 μm, or greater than 0.8 μm in ventral spinal cord (B,D). The distribution of axon diameters observed was unchanged at both P14 (A, B) and P40 (C,D). Numerical results are presented as mean ± SEM. (TIF) [file pbio.1002107.s004.tif]

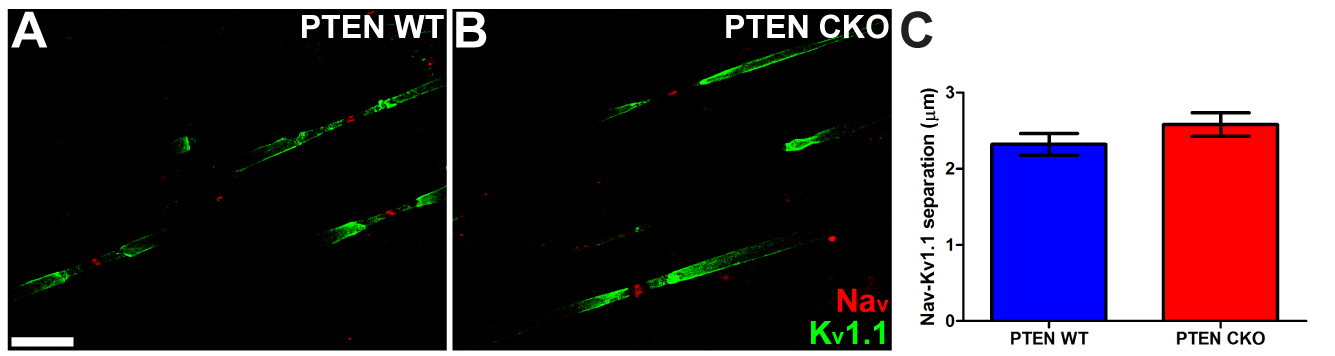

Supplement: S4 Fig — In teased ventral spinal cord preparations from both P40 PTEN cKO mice (B) and wild-type littermates (A), voltage-gated potassium channels were normally localised to the juxtaparanode (Kv1.1, green), with no change in the separation by paranodal junctions from voltage gated sodium channels at the node of Ranvier (Nav, red), indicating the paranodal barrier separating them remains intact (C). Numerical results are presented as mean ± SEM. At least 20 paranodes were analysed in five mice per genotype. Student's t test was used. Scale bar = 20 μm. (TIF) [file pbio.1002107.s005.tif]
